# Spatial Organization of the Gastrointestinal Microbiota in Urban Canada Geese

**DOI:** 10.1038/s41598-018-21892-y

**Published:** 2018-02-27

**Authors:** Sergei V. Drovetski, Michael O’Mahoney, Emma J. Ransome, Kenan O. Matterson, Haw Chuan Lim, R. Terry Chesser, Gary R. Graves

**Affiliations:** 1 Department of Vertebrate Zoology, National Museum of Natural History, Smithsonian Institution, Washington, DC 20004 USA; 2 Department of Invertebrate Zoology, National Museum of Natural History, Smithsonian Institution, Washington, DC 20004 USA; 3 Imperial College London, Silwood Park Campus, Ascot, SL5 7PY UK; 4 Consortium for the Barcode of Life, National Museum of Natural History, Smithsonian Institution, Washington, DC 20004 USA; 5 Department of Vertebrate Zoology, National Museum of Natural History & Center for Conservation Genomics, Smithsonian Institution, Washington, DC USA; 6 USGS Patuxent Wildlife Research Center, National Museum of Natural History, Smithsonian Institution, Washington, DC USA; 7 Center for Macroecology, Evolution and Climate, National Museum of Denmark, University of Copenhagen, DK-2100 Copenhagen Ø, Denmark; 8 Present Address: Department of Biology, George Mason University, Fairfax Va, USA

## Abstract

Recent reviews identified the reliance on fecal or cloacal samples as a significant limitation hindering our understanding of the avian gastrointestinal (gut) microbiota and its function. We investigated the microbiota of the esophagus, duodenum, cecum, and colon of a wild urban population of Canada goose (*Branta canadensis*). From a population sample of 30 individuals, we sequenced the V4 region of the 16S SSU rRNA on an Illumina MiSeq and obtained 8,628,751 sequences with a median of 76,529 per sample. These sequences were assigned to 420 bacterial OTUs and a single archaeon. *Firmicutes*, *Proteobacteria*, and *Bacteroidetes* accounted for 90% of all sequences. Microbiotas from the four gut regions differed significantly in their richness, composition, and variability among individuals. Microbial communities of the esophagus were the most distinctive whereas those of the colon were the least distinctive, reflecting the physical downstream mixing of regional microbiotas. The downstream mixing of regional microbiotas was also responsible for the majority of observed co-occurrence patterns among microbial families. Our results indicate that fecal and cloacal samples inadequately represent the complex patterns of richness, composition, and variability of the gut microbiota and obscure patterns of co-occurrence of microbial lineages.

## Introduction

The gastrointestinal tract (gut) of vertebrate animals, including birds, hosts a diverse community of *Bacteria*, *Archaea*, *Fungi*, and other eukaryotic microorganisms collectively termed microbiota^1^. Microbiotas profoundly affect the development, physiology, immune system, behavior, and reproductive success of their vertebrate hosts, and in a broader context, play an important role in vertebrate evolution^2^. Avian microbiome research is an exciting and rapidly developing field with evolving methods that have not yet been standardized. Recent reviews have identified a number of biases that hinder its progress: (*i*) high prevalence of captive animal studies, (*ii*) a focus on feces and the distal-most gut regions (colon or cloaca), (*iii*) small sample sizes, and (*iv*) poor taxonomic and geographic coverage of host species^2–5^.

The spatial and functional heterogeneity of microbiota observed along the gut^6–10^ raises concern about the degree to which fecal or cloacal samples adequately represent the richness and composition of the entire gut microbiota^5,8,11^. A recent study of eight captive Attwater’s prairie chickens (*Tympanuchus cupido attwateri*), which sampled the ileum, cecum, colon, and cloaca, demonstrated that the cloacal microbiota was similar to those of the ileum and colon but exhibited subtly different patterns of species richness and lineage abundance^8^. On the other hand, the cecal microbiota differed considerably from those of other gut regions^8^. Zhang *et al*.^8^ also noted large variation among individual prairie chickens in the bacterial communities of the ileum, colon, and cloaca, suggesting that large sample sizes are required for adequate characterization and comparison of microbiota among gut regions. Another recent study that compared microbiotas of the ileum, cecum, colon, cloaca, and feces in 20 juvenile ostriches (*Struthio camelus*) also demonstrated that cloacal and fecal samples were poorly representative of ileal and cecal microbiotas^11^.

In this paper we investigate the richness, composition (taxonomic membership and abundance), co-occurrence patterns, and variability of the gut *Bacteria* and *Archaea* (hereafter microbiota) within and among individuals of an urban population of the Canada goose (*Branta canadensis*). Resident populations of the Canada goose in North America grew rapidly from 250,000 in 1973 to almost 4,000,000 in 2012^12^. Dense aggregations of this species have become a nuisance in public parks, sports fields, golf courses, and other outdoor recreational areas by producing large quantities of feces, consuming turf, and for their aggressive behavior during the breeding season. Despite growing concerns about public health risks posed by resident Canada goose fecal contamination^13^ (but see^14^), only a single study has attempted to explore the fecal microbiota of this species beyond testing for a few potential human or domestic animal pathogens^15^. Lu *et al*.^15^ obtained a total of 749 16S SSU rRNA sequences from clone libraries of 39 fecal samples collected in Ohio (*n* = 12 geese), Oregon (*n* = 3), and Ontario (*n* = 24). They identified 73 unique haplotypes, which is considerably lower than the number of operational taxonomic units (OTUs) reported for other goose species in studies employing next generation sequencing of the 16S SSU rRNA. For example, two studies of the bar-headed goose (*Anser indicus*) identified 416 OTUs^16^ and 607 OTUs^17^ (97% similarity threshold), respectively, in just 9 and 6 fecal samples. A study of the domestic swan goose (*Anser cygnoides*) identified 2,359 OTUs in 26 fecal samples^18^. Although the number of reported OTUs depends on the clustering methodology, sequence quality and frequency filtering implemented in each study, these results suggest that the Canada goose microbiota remains largely unexplored. Understanding the composition of the gut community is also an important first step in monitoring and surveillance of zoonotic, emerging, agricultural, and piscicultural pathogens that may be transmitted by urban populations of Canada geese.

## Results

### Canada goose gut microbiota

Our initial dataset included 9,761,519 sequences collected from 120 samples (30 birds × 4 gut regions). We excluded a single cecal sample (AS508) due to a DNA extraction error. This exclusion combined with data filtering (see Methods) reduced the total number of sequences to 8,628,751. The number of sequences obtained from a single sample ranged from 5,387 to 154,650 with a median of 76,529.

Filtered sequences were assigned to 421 OTUs. A single OTU was classified as *Archaea (Euryarchaeota; Thermoplasmata; E2; [Methanomassiliicoccaceae]; vadinCA11*) whereas 420 OTUs were classified as *Bacteria*. All OTUs were classified to phylum and class, and all but a single OTU to order. The proportion of OTUs classified at lower taxonomic levels varied from 92.6% at the familial level, 53.7% at the generic level, to 7.8% at the specific level (Supplementary Table S1).

Among the 10 bacterial phyla identified in our dataset, *Firmicutes* was the most diverse (217 OTUs; Supplementary Table S1). *Proteobacteria* and *Bacteroidetes* were represented by 84 and 69 OTUs, respectively. Other phyla were less diverse: *Tenericutes* (16 OTUs), *Actinobacteria* (13 OTUs), *Cyanobacteria* and *Fusobacteria* (8 OTUs each), *Deferribacteres* and *Elusimicrobia* (2 OTUs each), and *Verrucomicrobia* (1 OTU). Bacterial OTUs were assigned to 48 families (Supplementary Table S1). This is a conservative estimate as we combined OTUs from unclassified families, as long as they belonged to the same order. The number of OTUs per family varied from 1 to 79. The *Ruminococcaceae* (*Firmicutes*) was the most diverse with 79 OTUs. *Lachnospiraceae* (*Firmicutes*) comprised 51 OTUs, *Pasteurellaceae* (*Proteobacteria*) 28 OTUs, *Bacteroidaceae* (*Bacteroidetes*) 27 OTUs, and *Clostridiaceae* and *Erysipelotrichaceae* (both *Firmicutes*) 19 OTUs each. The number of OTUs for each of the remaining families was < 15 (Supplementary Table S1).

At least six bacterial species and two genera in our dataset (*Clostridium perfringens*^19,20^, *Enterococcus cecorum*^21^, *Lactococcus garvieae*^22^, *Lawsonia intracellularis*^23^, *Neisseria sp*.^24^, *Riemerella anatipestifer*^25^, *Staphylococcus sp*.^26^, *Streptococcus* *suis*^27^) are known to include important human, avian, or other vertebrate pathogens.

### Structuring of microbiota among gut regions

**Figure 1:**
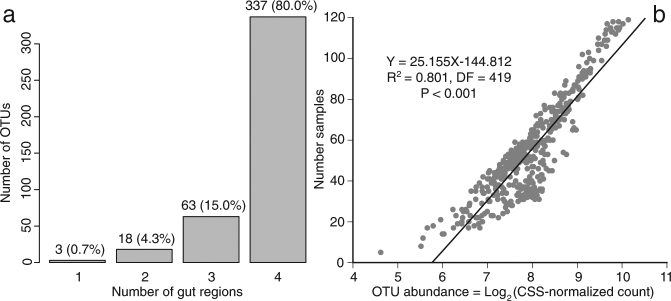
Number of OTUs observed in one, two, three, and all four gut regions (**a**) and the relationship between OTU abundance and the number of samples (esophagus, duodenum, caecum, and colon) in which an OTU was observed (**b**).

None of the OTUs had an overall abundance greater than 1% (Supplementary Table S1). Nonetheless, when all samples were considered together, 80% of OTUs (*n* = 337) had at least one representative sequence in all four gut regions, 15% (*n* = 63) in three regions, 4.3% (*n* = 18) in two regions, and only 0.7% (*n* = 3) in a single gut region (Fig. 1a). The connectivity among different gut regions was further supported by the presence of a positive abundance-occupancy relationship between the overall OTU abundance and the number of samples (esophagus, duodenum, cecum, and colon) in which they were detected (adjusted *r*^2^ = 0.801, df = 419, *P* < 0.0001; Fig. 1b). This relationship suggests that more abundant OTUs were detected in more samples regardless of which gut regions those samples come from.

**Figure 2:**
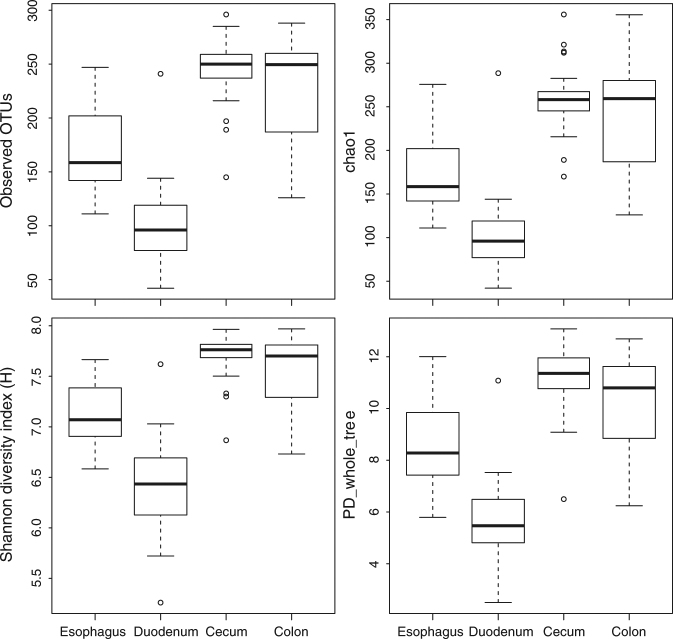
Comparison of selected microbial diversity indices among gut regions calculated using Log_2_-transformed CSS-normalized OTU abundance.

Notwithstanding the ubiquity of most OTUs, the four gut regions harbored distinctive microbial assemblages with regard to their richness, composition, and variability among individual birds. The duodenum harbored the most depauperate microbial community with an average of 101 ± 36 (mean ± standard deviation) OTUs per sample, whereas the cecum (264 ± 37 OTUs per sample) and colon (237 ± 58 OTUs per sample) harbored more than twice as many OTUs per sample (Fig. 2). The esophageal community showed intermediate richness (169 ± 39 OTUs per sample). Differences among the four gut regions in the number of OTUs per sample were significant (Mann-Whitney *U* Test: esophagus/duodenum: *Z* = 5.648; esophagus/cecum: *Z* = −5.693; esophagus/colon: *Z* = −4.132; duodenum/cecum: *Z* = −6.436; duodenum/colon: *Z* = −6.320; all *P* < 0.00001), except the difference between cecum and colon (*Z* = −0.516; *P* = 0.603). All calculated *α*-diversity indices showed similar patterns among gut regions (Supplementary Table S2). We found no difference in microbiota richness between the sexes in any of the gut regions (all Mann-Whitney *U* Test female/male: esophagus *Z* = 0.466, two-tailed *P* = 0.638; duodenum *Z* = −0.33867, *P* = 0.728; cecum *Z* = −1.860, *P* = 0.063; colon *Z* = 0.677, *P* = 0.497; Supplementary Fig. S1).

Also, gut regions differed in microbiota composition. Kruskal-Wallis tests with Bonferroni correction of *P*-values demonstrated that 91.4% (385 of 421) OTUs had significantly different abundances among the four gut regions (Supplementary Table S1). ADONIS indicated that differences among the four gut regions explained 67.7% of variance in the weighted UniFrac distance matrix (*df* = 3, F = 80.2, *r*^2^ = 0.677, *P* < 0.001) whereas effects of sex, age, individual or any variable interactions were not significant. In pairwise comparisons of gut regions, the esophageal community was the most distinctive. ADONIS *r*^2^-values for pairwise analyses of gastrointestinal regions that included esophagus ranged from 0.645 to 0.833: esophagus/duodenum: (*df* = 3, F = 105.6, *r*^2^ = 0.645, *P* < 0.001); esophagus/cecum (*df* = 1, F = 283.6, *r*^2^ = 0.833, *P* < 0.001); and esophagus/colon (*df* = 1, F = 156.4, *r*^2^ = 0.729, *P* < 0.001). The duodenal community was strongly differentiated from that of the cecum (ADONIS *df* = 1, F = 94.1, *r*^2^ = 0.623, *P* < 0.001) and colon (ADONIS *df* = 1, F = 43.0, *r*^2^ = 0.426, *P* < 0.001). The microbiota of the cecum and colon were the least distinctive among the four gut regions (ADONIS *df* = 1, F = 15.7, *r*^2^ = 0.216, *P* < 0.001).

**Figure 3:**
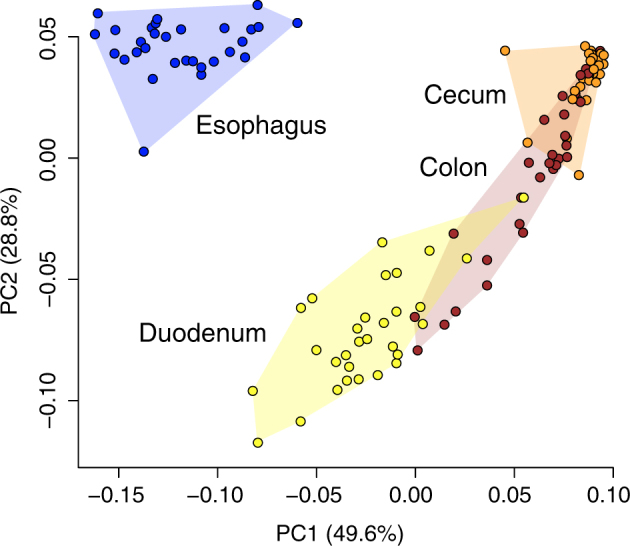
Plot of the Principal Coordinate Analysis (PCoA) of samples from different gut regions. Each circle represents one gut region sample from a single bird. Circles representing each gut region are connected by a minimum convex polygon.

**Figure 4:**
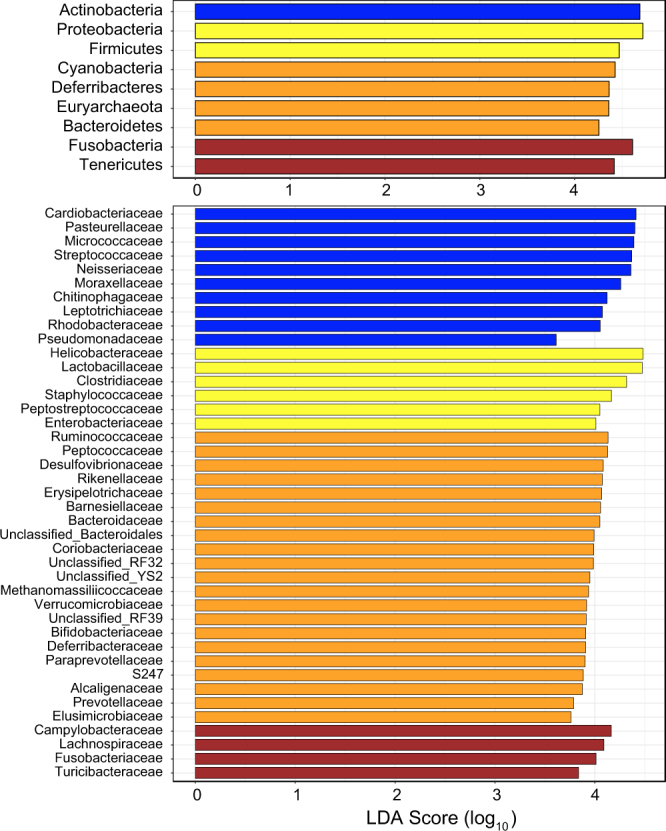
Results of the linear discriminant analysis (LDA) effect size (LEfSe) method for identification of phyla (top) and families (bottom) most responsible for the differences among the microbiotas of esophagus, duodenum, cecum, and colon. Color of the LDA score bar indicates the gut region: blue = esophagus, yellow = duodenum, orange = cecum, and brown = colon.

Our PCoA also indicated that esophageal, duodenal, and cecal microbiotas were strongly differentiated from one another (Fig. 3). Samples from these three gut regions formed non-overlapping clusters in the bivariate plot, with two axes that accounted for 49.6% (PC1) and 28.8% (PC2) of the variation. Colonic samples partially overlapped duodenal and cecal clusters. Although the three most abundant vertebrate gut bacterial phyla, *Firmicutes*, *Proteobacteria*, and *Bacteroidetes*^5^, accounted for ~90% of sequences in each of the gut regions, their relative abundance differed among these regions (Supplementary Table S1). The proportion of *Firmicutes* and *Bacteroidetes* increased distally along the gut from esophagus to cecum and colon, as the proportion of *Proteobacteria* decreased (Supplementary Fig. 2). The remaining eight phyla did not exhibit longitudinal succession along the gut but showed a peak abundance in single gut regions. The LEfSe method indicated that a high abundance of *Actinobacteria* contributed most to distinguishing esophageal microbiota from those of other gut regions (Fig. 4). *Proteobacteria* and *Firmicutes* contributed most to the distinctiveness of the duodenal community, whereas *Cyanobacteria*, *Deferribacteres*, *Euryarchaeota*, and *Bacteroidetes* appeared to be strongly associated with the cecum, and *Fusobacteria* and *Tenericutes* with the colon.

At the family level, the LEfSe method characterized differences among gut regions in greater detail (Fig. 4). Ten families representing five phyla contributed most to the differentiation of esophageal microbiota from those of other gut regions. Six families belonging to two phyla distinguished the duodenal microbiota. Twenty-one bacterial families representing 10 phyla distinguished the cecal microbiota, whereas colonic microbiota had only four strongly associated families representing three phyla.

Finally, the PCoA illustrated the differences in the variability among individuals of the cecal microbiota compared to those of other gut regions. The size of the cecal cluster in the PCoA plot (Fig. 3) was much smaller than the clusters formed by samples from other gut regions. The variance of PC1 scores for the cecal samples (1.157 × 10^−4^) was lower than the variance of the scores for the other three gut regions (esophagus 7.123 × 10^−4^; duodenum 8.114 × 10^−4^; colon 7.007 × 10^−4^). These differences were significant: cecum/esophagus (*F*_*(1, 57)*_ = 0.162, *P* = 6.561 × 10^−6^); cecum/duodenum (*F*_*(1, 57)*_ = 0.143, *P* = 1.644 × 10^−6^); and cecum/colon (*F*_*(1, 57)*_ = 0.165, *P* = 7.775 × 10^−6^). The variance of cecal PC2 scores (1.571 × 10^−4^) was also significantly lower than the variance of duodenal (5.060 × 10^−4^; *F*_(1, 57)_ = 0.311, *P* = 0.003) and colonic scores (1.271 × 10^−3^; *F*_(1, 57)_ = 0.130, *P* = 5.899 × 10^−7^). The variances of cecal (1.571 × 10^−4^) and esophageal (1.271 × 10^−4^) PC2 scores did not differ significantly (*F*_(1, 57)_ = 1.236, *P* = 0.573). Despite the high richness and evenness of cecal microbiotas (Fig. 2), they were the least variable among the gut regions in terms of both α and β diversity (Figs 2 and 3).

Microbiotas from different gut regions appear to vary independently within individual birds. Mantel tests of weighted UniFrac distance matrices for different gut regions showed no significant correlation (esophagus/duodenum *r* = 0.079, n = 29, *P* = 0.346; esophagus/cecum *r* = −0.153, n = 29, *P* = 0.157; esophagus/colon *r* = 0.025, n = 29, *P* = 0.726; duodenum/cecum *r* = −0.088, n = 29, *P* = 0.363; duodenum/colon *r* = 0.018, n = 29, *P* = 0.742; cecum/colon *r* = 0.084, n = 29, *P* = 0.239).

### Patterns of co-occurrence among microbial families

**Figure 5:**
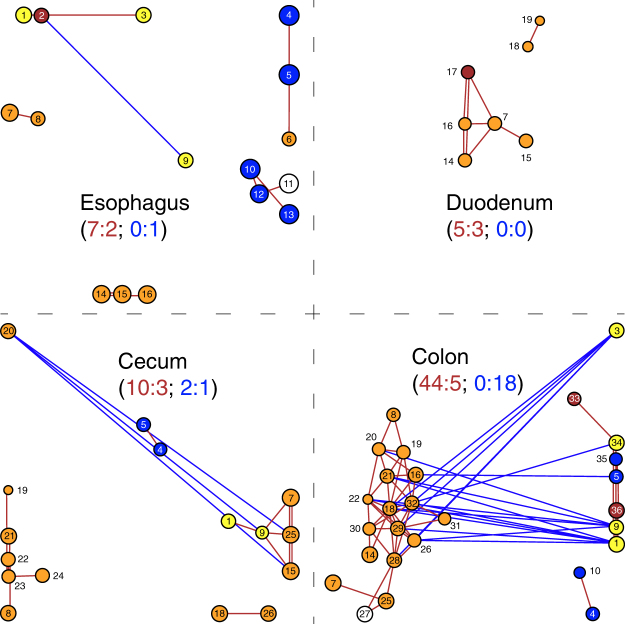
Association networks among families of Bacteria and Archaea in the gut regions. Taxa are represented as nodes whose location is determined by PCoA scores. CSS-normalized and Log_2_-transformed taxa abundance is shown as node size. The color of the circles indicates the gut region to which each family was assigned by the linear discriminant analysis (LDA) effect size (LEfSe) method (Fig. 4): blue = esophagus, yellow = duodenum, orange = ceca, brown = colon; white = family was not assigned to a gut region. Lines connecting nodes represent significant positive (red) or negative (blue) Spearman’s correlation between family abundances (i.e. co-occurrence patterns). Numbers next to or inside the nodes identify families: 1 - *Clostridiaceae*, 2 - *Fusobacteriaceae*, 3 - *Helicobacteraceae*, 4 - *Streptococcaceae*, 5 - *Pasteurellaceae*, 6 - *Deferribacteraceae*, 7 - *Ruminococcaceae*, 8 - unclassified *Mollicutes*, 9 - *Lactobacillaceae*, 10 - *Micrococcaceae*, 11 - *Weeksellaceae*, 12 - *Cardiobacteriaceae*, 13 - *Neisseriaceae*, 14 - *Paraprevotellaceae*, 15 - *Bacteroidaceae*, 16 - *Prevotellaceae*, 17 - *Lachnospiraceae*, 18 - *Rikenellaceae*, 19 - unclassified *Alphaproteobacteria*, 20 - unclassified *Cyanobacteria*, 21 - unclassified *Bacteroidales*, 22 – *[Methanomassiliicoccaceae]*, 23 - *Elusimicrobiaceae*, 24 - *Verrucomicrobiaceae*, 25 - *Erysipelotrichaceae*, 26 - *Barnesiellaceae*, 27 - *Veillonellaceae*, 28 - *Alcaligenaceae*, 29 - *Coriobacteriaceae*, 30 - *Peptococcaceae*, 31 - *Bifidobacteriaceae*, 32 - *Desulfovibrionaceae*, 33 - *Turicibacteraceae*, 34 - *Peptostreptococcaceae*, 35 - *Leptotrichiaceae*, 36 - *Campylobacteraceae*. Numbers in parentheses indicate ratios of positive (red) and negative (blue) abundance correlations between families associated with the same (left) and different (right) gut regions.

The number of significant correlations between abundances of microbial families was unequally distributed along the gut (Fig. 5). There were only 10 significant correlations (9 positive, 1 negative) in the esophagus and 8 significant correlations (all positive) in the duodenum. In the cecum, the number of significant correlations was notably higher (13 positive/3 negative), and the number of significant correlations in the colon was twice that of the other three gut regions combined (49 positive/18 negative).

Among 79 instances of significant positive correlations between abundances of microbial families (Fig. 5), 66 were between families that were assigned to the same gut region in our LEfSe results and only 13 were between families assigned to different gut regions (Fig. 4). This ratio was significantly different from equality (40/40; Fisher’s exact *P* = 9.204 × 10^−6^). In the cases of negative correlations, the pattern was reversed. Negative abundance correlations between families associated with the same gut region were observed only twice, whereas negative abundance correlations between families associated with different gut regions were observed 20 times. This ratio was also significantly different from equality (11/11; Fisher’s exact test *P* = 0.007).

## Discussion

Recent reviews of gut microbiota research on birds^2–4^ and other vertebrates^5^ have identified fundamental shortcomings. Use of captive animals fed artificial diets, inadequate sample sizes, and the nearly exclusive focus on fecal or cloacal samples likely underestimates the richness and composition of the gut microbiota and limits the scope of conclusions. Our study is the first to sample multiple gut regions in a free-living bird. The sampling of four functionally distinct gut regions in 30 individuals provides unprecedented insight on the regional structure of gut microbial communities and their variability among individuals.

The overall composition of the Canada goose gut microbiota appears similar to the fecal microbiota observed in an earlier study of this species^15^, and to the bar-headed goose^16,17^, a domestic form of the swan goose^18^, and to vertebrates in general^5^. *Firmicutes*, *Proteobacteria*, and *Bacteroidetes*, which dominate vertebrate gut microbiota, accounted for 90% of all sequences (Supplementary Fig. 2) and 88% of all OTUs in our dataset (Supplementary Table S1). *Tenericutes*, *Actinobacteria*, and *Fusobacteria*, which represented a group of less abundant and less diverse phyla (1.56%–3.59% of all sequences; 8–16 OTUs; Supplementary Fig. 2 and Supplementary Table S1), are typically found at low abundances in the vertebrate gut^5^. The remaining bacterial phyla, *Cyanobacteria* (1.03%, 8 OTUs), *Deferribacteres*, *Elusimicrobia*, and *Verrucomicrobia* (≤0.61%, ≤2 OTUs each; Supplementary Table S1), were also previously reported as low-abundance members of the fecal microbiota of the Canada, bar-headed or swan geese^16–18^. We refrain from in-depth comparisons with studies based on fecal samples of geese due to presence of significant spatial structuring of microbiota along the gut (see below).

Two recent reviews summarized data on bacterial pathogens that could be transmitted by several goose species in Europe and North America^14^ and specifically by urban Canada geese in the USA^13^. Although reaching opposite conclusions regarding the degree of potential threats posed by Canada geese in urban areas, both reviews emphasized the need for more detailed studies focused on *Escherichia coli, Chlamydia psittaci*, and members of genera *Campylobacter*, *Helicobacter*, and *Salmonella*. The urban Canada goose review^13^ also identified *Clostridium botulinum*, *Vibrio cholera*, *Pasteurella multocida*, *Yersinia sp*., *Brachyspira sp*., and *Borrelia sp*. as requiring further investigation.

Most of the studies considered in these reviews^13,14^ were relatively narrow in taxonomic scope, focusing on previously reported disease outbreaks. Our data are much broader in taxonomic scope. They allowed us to identify several emerging pathogens that also merit detailed investigation. For example, we detected high prevalence of *Clostridium perfringens* (100%), *Streptococcus suis* (100%) and *Staphylococcus sp*. (86.7%) in Canada geese. All three of these taxa include strains that cause serious human disease and *Streptococcus suis* is also a significant swine pathogen^19,20,26,27^. We detected a number of other potentially pathogenic taxa, such as *Enterococcus cecorum, Lactococcus garvieae, Lawsonia intracellularis, Neisseria sp*., and *Riemerella anatipestifer*, that include virulent strains that can cause serious infections in wild and domestic waterfowl, poultry, domestic mammals, and in pisciculture^21–25^.

Eighty percent of microbial OTUs occurred in all four gut regions and 15% were detected in three of the four gut regions (Fig. 1a). The positive abundance-occupancy relationship (Fig. 1b) suggests that microbiota exchange among gut regions was governed primarily by OTU abundance. More abundant OTUs were detected in a larger number of samples regardless of the gut region of these samples’ origin than were less abundant OTUs. The connectivity of gut regional microbiotas results from peristaltic movement of digesta through the gut. This movement is not strictly unidirectional in birds. Regular reflux pushes digesta from the ileum into the ventriculus^28^ and antiperistaltic movements transfer digesta from the colon into the ceca and the ileum^29^, resulting in bidirectional transport of microorganisms across gut regions.

Despite the observed connectivity, the microbiotas of the esophagus, duodenum, cecum, and colon differed in three important aspects: richness (Fig. 2), composition (Figs 3, 4, Supplementary Fig. 2), and variability among individuals (Fig. 3). Microbial richness of gut regions may be affected by their intrinsic environmental conditions. The duodenum reportedly provides the least hospitable environment for bacteria due to high concentration of enzymes and bile salts^29,30^ and its immediate location downstream from the “low pH filter” imposed by the ventriculus, where postprandial pH may be as low as 1.3–2.5 in carnivorous, piscivorous, and some omnivorous birds^28,31^. Concomitantly, we observed the lowest microbial richness in the duodenum (Fig. 2). In contrast, distal gut regions (cecum and colon), which are known to maintain low concentrations of antimicrobial compounds^29,30^, possessed more than twice the observed duodenal richness (Fig. 2). The esophagus exhibited intermediate microbial diversity (Fig. 2), perhaps owing to the reportedly low digestive activity^28–30^.

Significant variation in the microbiota composition of the esophagus, duodenum, cecum, and colon of the Canada goose (Fig. 3) may be determined by physiological differences among those gut regions^28–30,32,33^. The esophageal community had the most distinctive composition, perhaps due to higher oxygen availability than in other regions, low food digestive activity, and its position anterior to the low pH filter imposed by the ventriculus^28–30,32,33^. Significant differences between the duodenal and cecal microbiota composition (Fig. 3) are likely related to differences in available nutrients^28–30,32,33^. Colonic samples formed a cluster on our PCoA plot (Fig. 3) that fell between duodenal and cecal clusters. We speculate that the microbiota of the colonic samples was strongly affected by the relative proportions of digesta coming from the small intestine and cecum just prior to sampling (Fig. 4). The goose specimens were collected during the morning soon after the time when cecal contents are usually emptied^28,29^. This may explain why most of the colonic samples were more similar to cecal samples than to duodenal samples in our PCoA plot (Fig. 3). ADONIS also supported greater similarity between colonic and cecal microbiotas (*r*^2^ = 0.216, *P* < 0.001) than between colonic and duodenal microbiotas (*r*^2^ = 0.426, P < 0.001).

The variability of microbial richness (Fig. 2) and composition (Fig. 3) among individuals may be related to digesta passage rates. The cecum reportedly retains digesta for 12–35 hours in chicken^29,30^ and approximately 24 hours in wild grouse (Tetraoninae; S.V.D. personal observation), whereas digesta likely passes much more rapidly through other sampled gut regions^28–30^. Given a microbial generation time of approximately 30 min^34^, the cecal microbiota has an opportunity to stabilize richness and composition over dozens of generations before the contents are expelled. In contrast, the digesta retention time in other gut regions rarely exceeds a single microbial generation, making their microbiota transient. Our results showed that transient microbial communities of the esophagus, duodenum, and colon varied in their richness (Fig. 2) and composition (Fig. 3) among individual birds significantly more than the stable cecal microbiotas.

Regional microbiotas appeared to vary independently within individual geese. Mantel tests showed that differences among individuals in the microbiota of one gut region were not predictive of the differences among the same individuals in other gut regions. Independent variation of regional microbiotas may be related to the functional differences among gut regions that alter available nutrients, the physicochemical environment, and digesta retention times^28–30,35^. In turn, these differences result in different selection pressures favoring certain microbes and inhibiting others that overpower homogenizing effects of the connectivity among gut regions.

Significant abundance correlations between microbial families occurred more frequently in the distal gut regions (Fig. 5). The cecum displayed as many significant correlations as the esophagus and duodenum combined, whereas the colon exhibited twice as many significant correlations as the other three gut regions combined. Furthermore, positive correlations were significantly more frequent between families identified as biomarkers of the same regional (i.e., functional) communities. In contrast, negative correlations were significantly more frequent between families identified as biomarkers of different regional communities (Fig. 5). Downstream mixing of regional microbiotas in the goose gastrointestinal tract was likely responsible for much of the variation in correlation patterns among microbial families.

In conclusion, the results of this study provide empirical evidence supporting the recently voiced concerns^5,8,11^ that small sample sizes and sampling designs restricted to feces or to a single gut region do not adequately represent the complex patterns of richness, composition, and spatial structure of the gut microbiota. Inadequate sampling also obscures patterns of co-occurrence of microbial lineages, their association with gut anatomy, and the dynamics of physical mixing of digesta and microbial communities.

## Methods

### Goose microbiota sampling

This project was approved by the National Museum of Natural History’s (NMNH) Institutional Animal Care and Use Committee (IACUC; permit 2016–04) and all parts of this study have been conducted in accordance with the approved protocols and relevant guidelines and regulations. Canada goose specimens (*n* = 30) were salvaged on 6 July 2016 during population control activities conducted by APHIS Wildlife Services (United States Department of Agriculture) at a single small lake in the vicinity of Silver Spring, Montgomery County, Maryland. These included 4 recently fledged juvenile females, 4 females and 1 male of undetermined age (possibly > 1 year old), 10 adult females (>1 year of age), and 11 adult males (>1 year of age). Specimens were quickly chilled to a few degrees above 0 °C after euthanasia to avoid decomposition prior to sampling. Microbiome samples were taken within 3 hours of death.

We sampled microbiota in four gut regions: esophagus, duodenum, cecum, and colon^36^. We used sterile polyester-tipped applicators with 6 inch plastic shafts (Fisher Scientific, Hampton, NH, USA) to sample the esophagus. The applicators were inserted approximately 100 mm into the esophagus and gently rubbed against the epithelial lining. Immediately after sampling, applicator tips were placed in vials containing 1 mL of sterile RNAlater solution. To sample the other three gut regions, we isolated an approximately 25 mm segment of the intestine with two sterile surgical hemostats and injected the isolated section with 1 mL of HyPure molecular biology grade water (GE Healthcare Life Sciences, Logan, UT, USA) using a sterile single-use syringe. The water was pumped in and out of the syringe three times to ensure mixing with the intestinal contents. The resulting lavage mixture was immediately transferred into a collection vial containing 1 mL of RNAlater solution. We did not test experimentally whether swabbing and lavage methods recover different bacterial assemblages from the same gut regions. We assumed, however, that similar proportions of the common microbial OTUs would be represented in samples collected by both methods. The use of sterile materials and instruments in both sampling methods minimized the possibility of contamination. All sample vials were kept at room temperature for 24 hours to ensure mixing of the sample with RNAlater solution and then stored in an −80 °C freezer until DNA extraction. Voucher specimens were deposited in the National Museum of Natural History (USNM), Smithsonian Institution (catalog numbers, USNM 653216–653245).

### Molecular procedures

Prior to DNA extraction, we centrifuged the vials at 2 × 10^4^ G for 20 min and removed the RNAlater with a pipette using sterile filter tips. Total genomic DNA was extracted from the pellet formed at the bottom of the vial (duodenum, cecum, and colon) or from the pellet and applicator tip (esophagus) using the PowerSoil DNA Isolation Kit (Mo Bio Laboratories, Carlsbad, CA, USA) and then cleaned with the PowerClean Pro DNA Clean-Up Kit (Mo Bio Laboratories, Carlsbad, CA, USA). The clean-up step removes PCR inhibitors, and in this case, significantly improved amplification success and evenness of amplicon concentration, and eliminated smearing of amplicons on the agarose gel.

We followed the Earth Microbiome Project (http://www.earthmicrobiome.org) 16S SSU rRNA protocol^37^ for PCR amplification and sequencing. We amplified the V4 region of the 16S SSU rRNA using the primer pair 515FB and 806RB^38^. Each PCR contained 13 μL molecular grade PCR water, 10 μL 5 PRIME Hot Master Mix (5 PRIME Inc., Bethesda, MD, USA), 0.5 μL each of the forward and reverse primers (10 μM final concentration), and 10 ηg of genomic DNA. The PCR profile included an initial denaturation at 94 °C for 3 min, 35 cycles of amplification (45 s at 94 °C, 60 s at 50 °C, 90 s at 72 °C), and a final extension of 10 min at 72 °C.

Each sample was amplified in triplicate. A negative control PCR containing 1 μL sterile water was set up for each triplicate. PCR products were checked on a 1.5% agarose gel. The triplicates were combined and their pooled amplicon concentration was determined using a Qubit 2.0 fluorometer with the dsDNA High Sensitivity Assay Kit (Life Technologies Corp., Carlsbad, CA, USA). Approximately 240 ng of each combined triplicate was pooled into a single library, which was cleaned with the UltraClean PCR Clean-Up Kit (Mo Bio Laboratories, Carlsbad, CA, USA), and normalized to 2 nM concentration. The resultant library was sequenced on an Illumina MiSeq using the 300-cycle MiSeq Reagent Kit v2 (Illumina, Inc., San Diego, CA, USA) according to the Earth Microbiome Project protocol^38^. The length of the complete 16S SSU rRNA fragment varied between 252 and 254 bp, with most sequences being 253 bp. This is the standard length of 16S SSU rRNA fragments used by the Earth Microbiome Project for surveys of microbial communities^38^ and it considerably exceeds the sequence length (100 bp) demonstrated to be sufficient for resolving differences among microbial communities^39^.

### Illumina data processing

Raw forward and reverse reads were joined (join_paired_ends.py), demultiplexed (split_libraries_fastq.py), and quality filtered (quality score Q ≥ 30, maximum barcode error = 0, minimum length = 200 bp) using the Quantitative Insights Into Microbial Ecology (QIIME) pipeline v 1.9.0^40^.

Chimeric sequences were identified using the USEARCH algorithm^41^ against the ChimeraSlayer reference database (identify_chimeric_seqs.py; version ‘microbiomeutil-r20110519’) and subsequently removed from the dataset (filter_fasta.py) prior to sequence alignment. Sequences were aligned with the SILVA 16S SSU rRNA reference database^42^ and clustered into OTUs at 3% divergence^42,43^. OTUs were taxonomically classified against the SILVA v123 16S database^43^ and compiled at each taxonomic level into OTU tables.

We filtered out singleton sequences, as well as any sequences classified as *Mitochondria*, *Eukaryota*, *Chloroplast*, or of unknown origin (filter_OTUs_from_otu_table.py). We also filtered out OTUs that had an overall relative abundance < 0.01% (filter_OTUs_from_otu_table.py) to reduce the likelihood of spurious OTUs affecting downstream diversity metrics^44^. OTU abundances were cumulative sum scaled (CSS)^45^ and Log_2_-transformed in Calypso 8.10^46^ to account for variation in sequencing depth among samples^47^ and non-normal distribution of abundances among OTUs. All data analyses were based on CSS + Log_2_-normalized OTU abundances.

### Data analyses

To ensure our sequencing coverage was sufficient, we constructed a rarefaction plot for all our samples in Calypso (Supplementary Fig. 3). Calypso was also used for the linear discriminant analysis effect size (LEfSe) method^48^ to identify microbial families that explain microbiota differences among gut regions (i.e. biomarker discovery). LEfSe is a three-step algorithm that first conducts a factorial Kruskal-Wallis sum-rank test for each family to identify those with significantly different abundances among gut regions. It then uses a set of pairwise Wilcoxon rank-sum tests to select a subset of differentially abundant families associated with a single gut region. Lastly, LEfSe uses a linear discriminant analysis to estimate the effect size of each differentially abundant family. To identify significant co-occurrence patterns among bacterial families within each gut region we constructed correlation networks in Calypso. Networks are built by computing Spearman’s rho values for correlations between taxa abundances. These values were then converted into dissimilarities used in Principal Coordinate Analysis (PCoA) to determine node positions in a two-dimensional plot. To simplify the presentation in Fig. 5, we deleted nodes with no statistically significant correlations (*P* > 0.05).

We used R version 3.3.3 (http://www.R-project.org) to generate plots and conduct regression analysis, Mann-Whitney *U, F*, and Fisher’s exact tests. To test the abundance-occupancy relationship, we conducted a regression analysis of the number of samples containing OTUs versus their abundance. We used Mann-Whitney *U* tests to compare richness among gut regions. To compare variance of the Principal Coordinate Analysis (PCoA) scores among gut regions, we conducted pairwise *F*-tests. We used Fisher’s exact test to determine whether the ratio of significant correlations between abundances of the families associated with the same or different gut regions differed from equality.

We used QIIME for the following α and β-diversity analyses. To compare microbiota richness and evenness among gut regions and between sexes we calculated all available *α*-diversity indexes (alpha_diversity.py). We used the Kruskal-Wallis test (group_significance.py) to test for differential abundance of OTUs among gut regions. To compare microbiota composition among gut regions and between sexes within each gut region, we calculated weighted UniFrac distances^49^ among samples and conducted PCoA (beta_diversity_through_plots.py). The weighted UniFrac distances were also used in the Permutational Multivariate Analysis of Variance Using Distance Matrices (ADONIS)^50^ implemented in R package Vegan 2.4–4^51^. Finally, we used the Mantel test (compare_distance_matrices.py) to test whether differences in microbiota composition among individual geese in one gut region were related to the differences in other gut regions. We used the significance level α = 0.05 for all statistical tests.

## Electronic supplementary material

Supplementary Information

Supplementary Table S1

Supplementary Table S2
