# Publisher Correction: Spatial Organization of the Gastrointestinal Microbiota in Urban Canada Geese

**DOI:** 10.1038/s41598-018-24859-1

**Published:** 2018-04-25

**Authors:** Sergei V. Drovetski, Michael O’Mahoney, Emma J. Ransome, Kenan O. Matterson, Haw Chuan Lim, R. Terry Chesser, Gary R. Graves

**Affiliations:** 1 Department of Vertebrate Zoology, National Museum of Natural History, Smithsonian Institution, Washington, DC 20004 USA; 2 Department of Invertebrate Zoology, National Museum of Natural History, Smithsonian Institution, Washington, DC 20004 USA; 3 Imperial College London, Silwood Park Campus, Ascot, SL5 7PY UK; 4 Consortium for the Barcode of Life, National Museum of Natural History, Smithsonian Institution, Washington, DC 20004 USA; 5 Department of Vertebrate Zoology, National Museum of Natural History & Center for Conservation Genomics, Smithsonian Institution, Washington, DC USA; 6 USGS Patuxent Wildlife Research Center, National Museum of Natural History, Smithsonian Institution, Washington, DC USA; 7 Center for Macroecology, Evolution and Climate, National Museum of Denmark, University of Copenhagen, DK-2100 Copenhagen Ø, Denmark; 8 Present Address: Department of Biology, George Mason University, Fairfax, Va USA

Correction to: *Scientific Reports* 10.1038/s41598-018-21892-y, published online 27 February 2018

In this Article, the Data availability section was omitted from the Additional Information section:

Data availability: The raw sequences archive has been deposited to GenBank: BioProject ID PRJNA396973 http://www.ncbi.nlm.nih.gov/bioproject/396973.

