# Supplementary material for: Spatial Organization of the Gastrointestinal Microbiota in Urban Canada Geese
Source: Sci Rep. 2018 Feb 27;8:3713. doi: 10.1038/s41598-018-21892-y (PMC5829075; doi:10.1038/s41598-018-21892-y)

Supplementary information for the study

SPATIAL ORGANIZATION OF THE GASTROINTESTINAL MICROBIOTA IN URBAN CANADA GEESE

Sergei V. Drovetski[1*], Michael O'Mahoney[2], Emma J. Ransome[3], Kenan O. Matterson[4], Haw Chuan Lim[5, 6], R. Terry Chesser[7], Gary R. Graves[1, 8]

[1] - Department of Vertebrate Zoology, National Museum of Natural History, Smithsonian Institution, Washington, DC, USA (* corresponding author e-mail: sdrovetski@gmail.com)

[2] - Department of Invertebrate Zoology, National Museum of Natural History, Smithsonian Institution, Washington, DC, USA

[3] - Imperial College London, Silwood Park Campus, Buckhurst Road, Ascot, UK

[4] - Consortium for the Barcode of Life, National Museum of Natural History, Smithsonian Institution, Washington, DC, USA

[5] - Department of Vertebrate Zoology, National Museum of Natural History & Center for Conservation Genomics, Smithsonian Institution, Washington, DC, USA.

[6] - Current Address: Department of Biology, George Mason University, Fairfax Va, USA

[7] - USGS Patuxent Wildlife Research Center, National Museum of Natural History, Washington, DC, USA

[8] - Center for Macroecology, Evolution and Climate, National Museum of Denmark, University of Copenhagen, DK-2100 Copenhagen Ø, Denmark.

Supplementary Table S1. List of OTUs, their taxonomy, results of the Kruskal–Wallis test, OTU CSS-normalized and Log_2-transformed abundance for each sample and its mean for each gut region. The letters after sample IDs indicate the gut region: e = esophagus, d = duodenum, ce = cecum, co = colon.

Supplementary Table S2. Microbial α-diversity indexes for individual samples and gut regions.

Supplementary Figure S1. Sex-specific microbiota richness in different gut regions

Supplementary Figure S2. Microbiota composition at the phylum level at four gut regions.

Supplementary Figure S3. Rarefaction plots for individual samples.

Supplementary Figure S1. Sex-specific microbiota richness in different gut regions

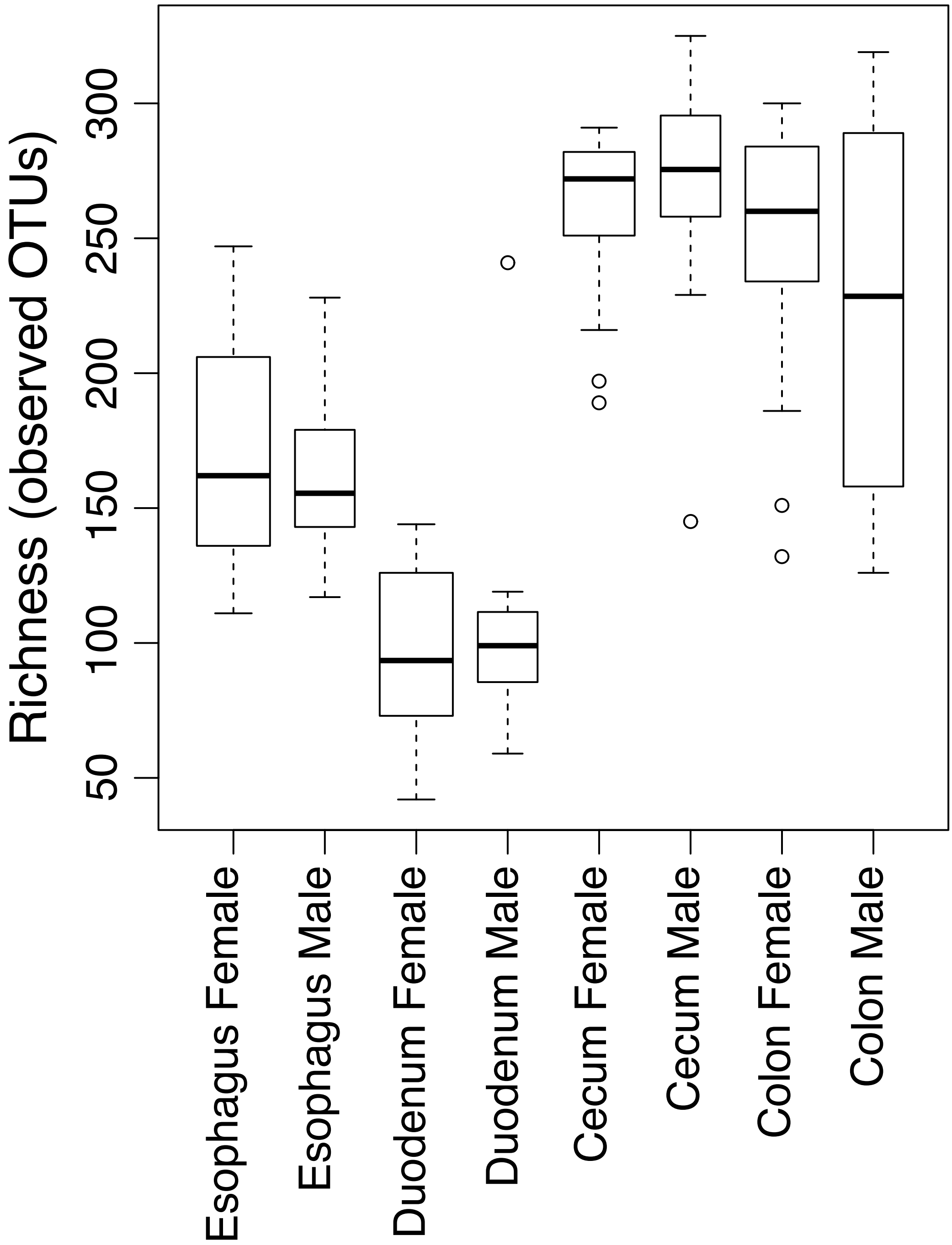

Supplementary Figure S2. Microbiota composition at the phylum level at four gut regions

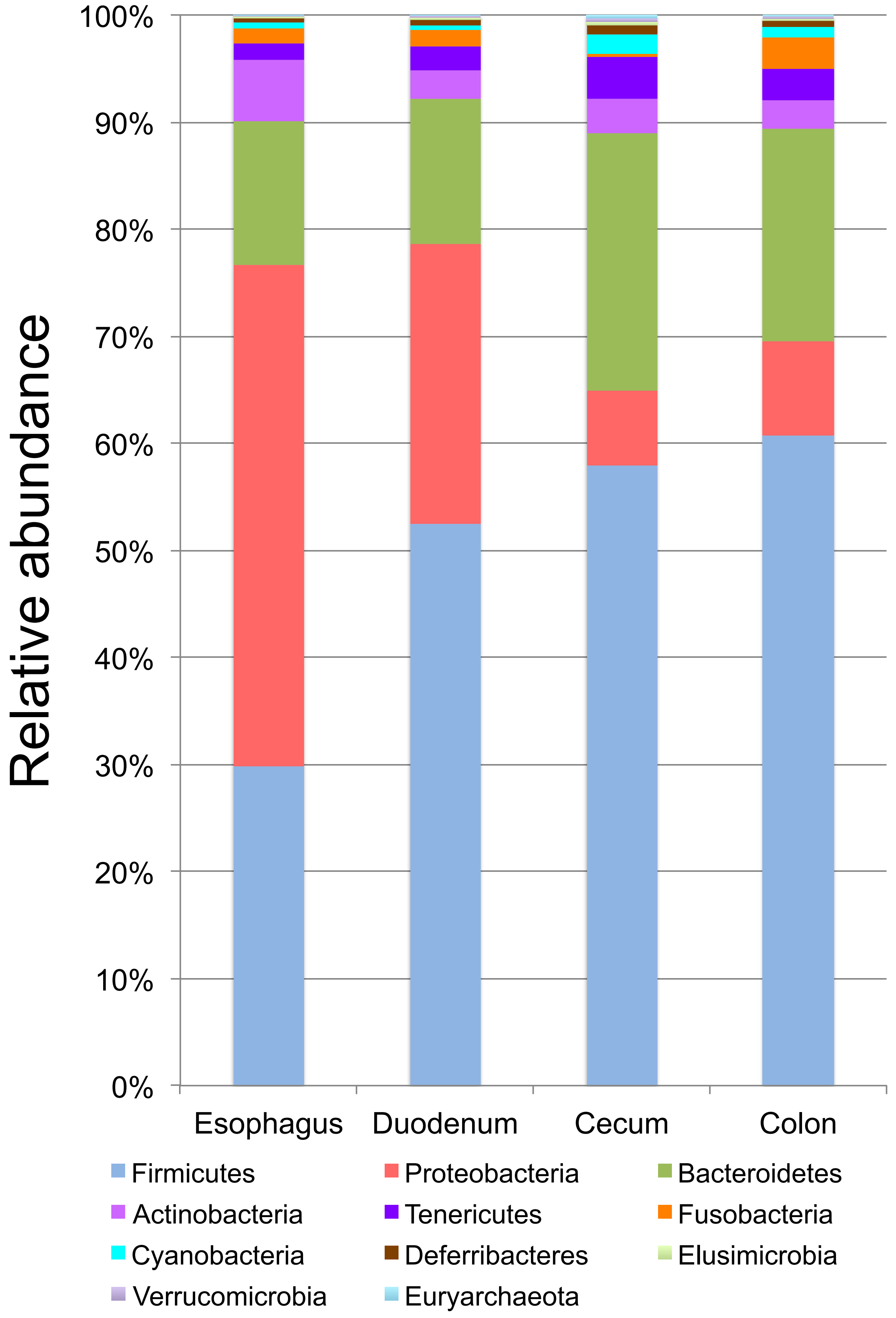

Supplementary Figure S3 Rarefaction plots for individual samples

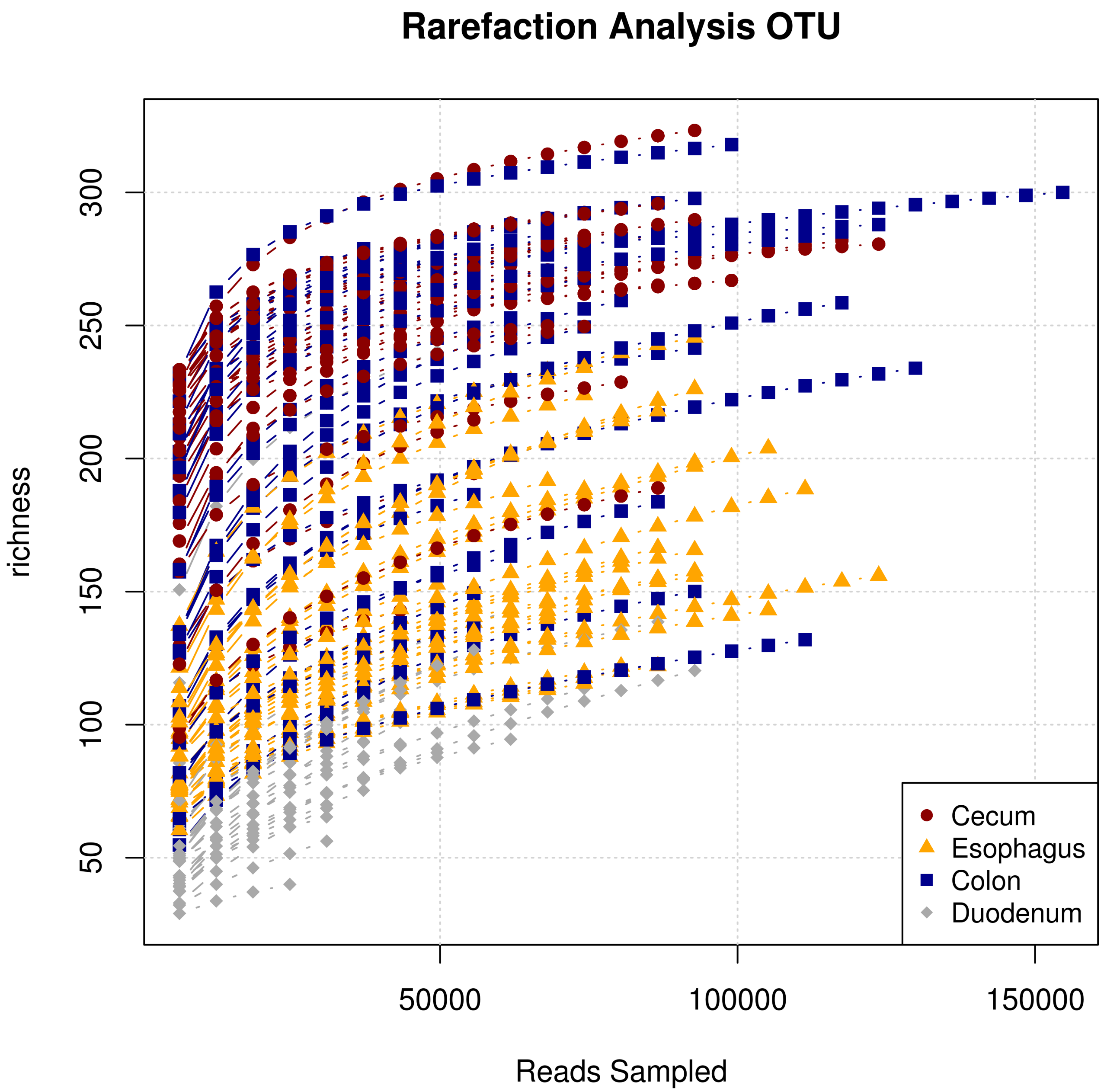

Supplement: Supplementary file 1 — Supplementary Information [file 41598_2018_21892_MOESM1_ESM.pdf]
